# Interface-Strengthened Ru-Based Electrocatalyst for High-Efficiency Proton Exchange Membrane Water Electrolysis at Industrial-Level Current Density

**DOI:** 10.3390/ma17204991

**Published:** 2024-10-12

**Authors:** Wenjun Lei, Xinxin Zhao, Chao Liang, Huai Wang, Xuehong Li, Mingkun Jiang, Xiaofeng Li, Fengqin He, Yonghui Sun, Gang Lu, Hairui Cai

**Affiliations:** 1 Qinghai Upstream of the Yellow River Hydropower Development Co., Ltd., Photovoltaic Industry Technology Branch Company, State Power Investment Corporation, Photovoltaic (Energy Storage) Industry Innovation Center, Photovoltaic Technology Research and Development Department, No. 399 South Yanta Road, Xi’an 710000, China; wenjun_lei2017@163.com (W.L.); xinxinzhao0508@163.com (X.Z.); lxh676@126.com (X.L.); jiangmk_spic@163.com (M.J.); lxf_ldj@163.com (X.L.); fq_he@126.com (F.H.); yonghuisun0724@163.com (Y.S.); 2 MOE Key Laboratory for Non-Equilibrium Synthesis and Modulation of Condensed Matter, Key Laboratory of Shaanxi for Advanced Materials and Mesoscopic Physics, State Key Laboratory for Mechanical Behavior of Materials, School of Physics, Xi’an Jiaotong University, No. 28 West Xianning Road, Xi’an 710049, China; chaoliang@xjtu.edu.cn (C.L.); wanghuaialso@stu.xjtu.edu.cn (H.W.)

**Keywords:** interfacial electronic effect, oxygen vacancy, RuO_2_, Nb_2_O_5_, PEMWE

## Abstract

Developing an OER electrocatalyst that balances high performance with low cost is crucial for widely adopting PEM water electrolyzers. Ru-based catalysts are gaining attention for their cost-effectiveness and high activity, positioning them as promising alternatives to Ir-based catalysts. However, Ru-based catalysts can be prone to oxidation at high potentials, compromising their durability. In this study, we utilize a simple synthesis method to synthesize a SnO_2_, Nb_2_O_5_, and RuO_2_ composite catalyst (SnO_2_/Nb_2_O_5_@RuO_2_) with multiple interfaces and abundant oxygen vacancies. The large surface area and numerous active sites of the SnO_2_/Nb_2_O_5_@RuO_2_ catalyst lead to outstanding acidic oxygen evolution reaction (OER) performance, achieving current densities of 10, 50, and 200 mA cm^−2^ at ultralow overpotentials of 287, 359, and 534 mV, respectively, significantly surpassing commercial IrO_2_. Moreover, incorporating Nb_2_O_5_ into the SnO_2_/Nb_2_O_5_@RuO_2_ alters the electronic structure at the interfaces and generates a high density of oxygen vacancies, markedly enhancing durability. Consequently, the membrane electrode composed of SnO_2_/Nb_2_O_5_@RuO_2_ and commercial Pt/C demonstrated stable operation in the PEM cell for 25 days at an industrial current density of 1 A cm^−2^. This research presents a convenient approach for developing a highly efficient and durable Ru-based electrocatalyst, underscoring its potential for proton exchange membrane water electrolysis.

## 1. Introduction

The intermittency and volatility of renewable energy sources such as wind and solar pose significant challenges to their integration into large-scale grid-connected power generation systems [1]. Proton exchange membrane water electrolysis (PEMWE) hydrogen production technology features a compact structure, rapid response, high current density, and high product purity, providing a flexible and efficient way to store renewable electricity in the form of hydrogen [2,3].

The PEM technique encompasses two primary half-reactions: the hydrogen evolution reaction (HER) at the cathode and the oxygen evolution reaction (OER) at the anode [4]. While the HER is generally more straightforward in PEMWE, the OER presents challenges due to its slower kinetics and higher energy barriers, thereby hindering efficient hydrogen production [5,6]. Additionally, the efficiency of this process is primarily limited by the catalytic activity and durability of the OER catalysts. Precious metals like iridium black and iridium oxide are predominantly used as commercial PEM anode catalysts due to their robustness and effectiveness [7]. However, Ir-based catalysts’ high cost and limited availability constrain the large-scale application of PEMWE [8,9]. Therefore, efficient and durable alternatives to iridium for acidic OER electrocatalysts are urgently needed.

Due to its high pseudopotential, rutile ruthenium dioxide (RuO_2_) is also recognized as a benchmark catalyst similar to IrO_2_ for the acidic OER [10]. However, RuO_2_ is prone to oxidation at anodic potentials, which causes catalyst deactivation [11,12]. It has been reported that integrating metal oxides with RuO_2_ to create heterostructures can effectively redistribute electrons, leverage synergistic effects, and, thereby, prevent the dissolution of Ru while enhancing catalytic activity and durability [13,14]. For instance, through constructing the RuO_2_/CoOx catalyst, the sacrificial oxidation of the CoOx substrate and the electron interaction at the Ru-O-Co interface enhances stability. Simultaneously, the exposed Ru/Co bimetallic sites around the interface achieve high activity and stability for RuO_2_ [15]. The interface in the RuO_2_/MnO_2-γ_ heterostructure enables MnO_2-γ_ to generate more reactive oxygen defects, facilitating electron transfer. Additionally, it helps mitigate peroxidation reactions and structural degradation of RuO_2_ in acidic environments, thereby suppressing the lattice oxygen mechanism (LOM) and enhancing stability [16]. In summary, the interface between RuO_2_ and metal oxides creates new active sites that stabilize RuO_2_. Furthermore, defects occurring during the formation of heterogeneous structures can optimize the catalyst’s electronic structure, thus enhancing the performance of the OER.

During actual PEMWE testing, industrial-scale PEM cells typically operate at higher current densities (exceeding 1 A cm^−2^) [17]. This necessitates electrode materials with highly efficient catalytic sites, rapid charge and mass transport rates, and strong adhesion to endure intense bubble generation challenges [18]. However, numerous electrocatalysts developed in laboratories exhibit robust performance at small scales or low current densities but struggle under high current density conditions at industrial scales, primarily due to challenges such as inert mass transport and the deactivation of active catalyst components [19]. Therefore, identifying suitable metal oxides to combine with RuO_2_, understanding their mechanisms comprehensively, and optimizing their structural and compositional benefits are essential for achieving efficient PEMWE operation at industrial-scale current densities. Studies have shown that niobium pentoxide (Nb_2_O_5_) has a strong REDOX ability and Lewis acid site, which facilitates the strong interaction with RuO_2_ [20], and, thus, plays an important role in regulating the intrinsic activity of OER active species and stabilizing catalysts [21].

Herein, we synthesize a SnO_2_, Nb_2_O_5_, and RuO_2_ composite catalyst (SnO_2_/Nb_2_O_5_@RuO_2_) using the Adams fusion method, featuring multiple interfaces and rich oxygen vacancy. Due to its large surface area and abundant active sites, the SnO_2_/Nb_2_O_5_@RuO_2_ catalyst exhibits outstanding acidic oxygen evolution reaction (OER) activity. It achieves current densities of 10, 50, and 200 mA cm^−2^ at ultralow overpotentials of 287, 359, and 534 mV, respectively, demonstrating excellent three-electrode stability. Importantly, the lattice oxygen mechanism of SnO_2_/Nb_2_O_5_@RuO_2_ is effectively modulated under the influence of multiple interface effects and oxygen vacancies, suppressing the dissolution of RuO_2_. Consequently, SnO_2_/Nb_2_O_5_@RuO_2_ membrane electrode assembly, in combination with commercial Pt/C, shows sustained stability under industrial current density (1 A cm^−2^) in PEM electrolysis cells for up to 25 days.

## 2. Materials and Methods

### 2.1. Chemicals and Materials

Stannous chloride (SnCl_2_·H_2_O), ruthenium trichloride (RuCl_3_), niobium chloride (NbCl_5_), sodium nitrate (NaNO_3_), and commercial iridium oxide (IrO_2_) were purchased from Aladdin Reagent Co., Ltd. (Shanghai, China). Ethanol (C_2_H_6_O), isopropanol (C_3_H_8_O), hydrochloric acid (HCl), and sulfuric acid (H_2_SO_4_) were sourced from Sinopharm Chemical Reagent Co., Ltd. (Beijing, China). Proton exchange membrane (PEM) was obtained from Chemours Technology Co., Ltd. (Wilmington, DE, USA). Nafion solution (5 wt%) was acquired from Sigma-Aldrich Co., Ltd. (St. Louis, MO, USA). The commercial Pt/C (20 wt%, Vulcan XC-72) was purchased from Johnson Matthey Reagent Co., Ltd. (London, UK). All chemical reagents were used as received without further purification.

### 2.2. Preparation of the SnO_2_

To begin, 10 g of SnCl_2_·H_2_O was weighed and added to a three-necked flask containing 50 mL of ethanol and 50 mL of isopropanol. The flask was then transferred to an oil bath and maintained at 60 °C with stirring for 1 h. Next, 100 g of NaNO_3_ was weighed and added to the flask. The oil bath temperature was increased to 80 °C, and stirring continued for 3 h. Subsequently, the reaction solution was transferred to a beaker and dried overnight at 80 °C in an oven. Finally, the dried precursor was placed in a corundum crucible and moved to a muffle furnace. The temperature was raised to 500 °C and sintered for 2 h, followed by natural cooling to room temperature. The sample was stirred in 0.2 M HCl for 1 h and underwent 5–10 rounds of filtration and washing. The washed sample was dried in an oven to obtain the SnO_2_ support.

### 2.3. Preparation of the SnO_2_@RuO_2_ and SnO_2_/Nb_2_O_5_@RuO_2_ Catalyst

Initially, 500 mg of preprepared SnO_2_ support was weighed and added to a three-necked flask containing 15 mL of ethanol and isopropanol. After ultrasonic dispersion, the flask was transferred to an oil bath at 60 °C and stirred for 1 h. Next, 0.5 mL of a 0.5 M RuCl_3_ solution was added to the flask. The temperature was then raised to 80 °C, and stirring continued for 3 h. The resulting solution was transferred to a beaker and dried overnight at 80 °C in an oven. Finally, the dried precursor was placed in a corundum crucible and transferred to a muffle furnace. The temperature was raised to 350 °C and sintered for 2 h, followed by natural cooling to room temperature. The sample was removed, stirred in 0.2 M HCl for 1 h, and then subjected to 5–10 rounds of filtration and washing. The washed sample was dried in an oven to obtain the SnO_2_@RuO_2_ catalyst. The synthesis method of SnO_2_/Nb_2_O_5_@RuO_2_ is essentially the same as that of SnO_2_@RuO_2_, with the only difference being the addition of an extra 73 mg of NbCl_5_.

### 2.4. Electrochemical Measurements

Preparation of the working electrode: 10 mg of catalyst was weighed and added to a serum bottle. A mixture of isopropanol, water, and Nafion (in a volume ratio of 2:1:0.05) totaling 5 mL was added, and the mixture was sealed and subjected to 30 min of ultrasonication to form a uniform ink-like electrode suspension. Afterward, 18.5 μL of the electrode suspension was drop-cast onto the surface of a rotating disk electrode, resulting in a final measured working electrode density of 0.3 mg/cm^2^.

The electrochemical properties of the samples were investigated using a CHI 760E electrochemical workstation from Shanghai Chenhua Technology Co., Ltd. (Shanghai, China), employing a three-electrode system. The catalyst drop-cast onto a glassy carbon rotating disk electrode (RDE) (0.196 cm^2^) as the working electrode. The carbon rod and saturated calomel electrodes (SCE) served as the counter and reference electrodes. All the electrochemical measurements were tested in the 0.5 M H_2_SO_4_ solution. All electrochemical potentials were converted relative to the reversible hydrogen electrode following the Nernst equation: E_(vs. RHE)_ = E_(vs. SCE)_ + 0.0591 × pH [22]. Before electrochemical testing, the test solution underwent a 25-min N_2_ purge to achieve saturation and eliminate dissolved O_2_ from the electrolyte. The linear sweep voltammetry (LSV) tests were performed at a scan rate of 5 mV/s with 90% iR compensation. The electrochemical impedance spectrum (EIS) was carried out from 10^−2^ to 10^5^ Hz with an amplitude of 5 mV, and the voltage of Nyquist diagrams was 1.0 V (vs. RHE). The PEMWE performance was assessed using Dalian RIGOR’s 500 W PEM testing rig (Dalian, China). The PEM cell was activated for 2 h at a voltage of 1.5 V, using a peristaltic pump at a rate of 20 rpm/min (controls the flow rate of water), and maintained a temperature of 60 °C. During the chronopotentiometry stability measurement, the voltage change of the PEM cell was recorded under a constant current of 25 A (resulting in a current density of 1 A/cm^2^), with the peristaltic pump operating at 40 rpm and at a temperature of 60 °C.

### 2.5. Proton Exchange Membrane Pretreatment Method

To remove organic impurities from the surface of the proton exchange membrane, the Nafion membrane was immersed in a beaker containing a 3% H_2_O_2_ solution and maintained at 90 °C for 1 h. The Nafion membrane was then thoroughly rinsed with pure water to eliminate residual H_2_O_2_. Next, the membrane was placed in a clean beaker with a 5% H_2_SO_4_ solution and kept at 90 °C for an additional hour. Afterward, the Nafion membrane was washed repeatedly with ultrapure water to remove any remaining H_2_SO_4_ and subsequently placed in a constant-temperature water bath at 80 °C for 15 min. Finally, the pretreated Nafion membrane was sealed in ultrapure water using cling film for storage.

### 2.6. Preparation of Membrane Electrode Assembly (MEA)

Membrane electrode slurry: The cathode catalyst utilized a 50 wt% Pt/C catalyst, while the anode employed our catalyst (SnO_2_/Nb_2_O_5_@RuO_2_). We began by adding the catalyst powder to a mixture of isopropanol and water in a volume ratio of 9:1, followed by incorporating the Nafion solution. The configurations were as follows: the Pt/C slurry was approximately 1 mg/mL, and the anode SnO_2_/Nb_2_O_5_@RuO_2_ catalyst was about 10 mg/mL. The slurry was ultrasonically dispersed in an ultrasonic cleaner for 5 h to ensure uniform dispersion.

Spraying: During the spraying process, the application amount was determined based on the desired noble metal content, as specified: 0.5 mg (Pt)/cm^2^ for the cathode and 0.1 mg (Ru)/cm^2^ for the anode.

Hot pressing: The membrane electrode was processed in a hot press under the following conditions: a pressure of 0.4 MPa and a temperature of 135 °C for 60 s.

## 3. Results and Discussion

### 3.1. Characterization of the Catalysts

**Figure 1:**
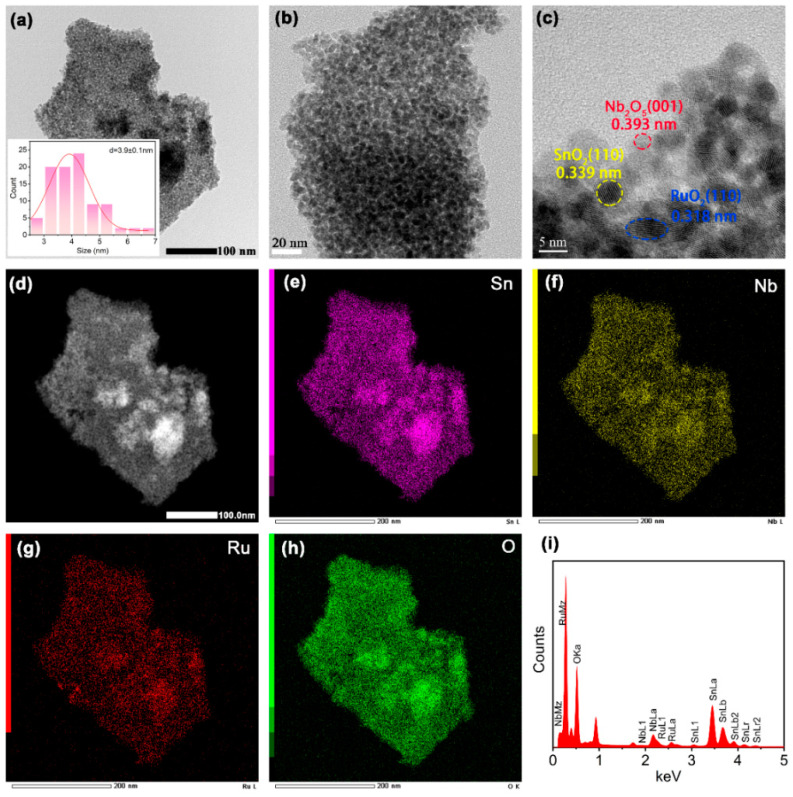
(**a**,**b**) TEM images of SnO_2_/Nb_2_O_5_@RuO_2_ at different magnifications; (**c**) HRTEM image of SnO_2_/Nb_2_O_5_@RuO_2_; (**d**) HAADF-STEM image of SnO_2_/Nb_2_O_5_@RuO_2_; (**e**–**h**) the elemental mappings of Sn, Nb, Ru, and O for SnO_2_/Nb_2_O_5_@RuO_2_; (**i**) EDX analysis of SnO_2_/Nb_2_O_5_@RuO_2_.

Figure 1a,b display TEM images of SnO_2_/Nb_2_O_5_@RuO_2_ at different magnifications, illustrating a highly uniform nanoparticle structure with an average diameter of approximately 3.9 ± 0.1 nm (Figure 1a insert). In addition, the HRTEM image in Figure 1c reveals lattice fringes measuring 0.393 nm, 0.339 nm, and 0.318 nm, corresponding to the crystal planes Nb_2_O_5_ (001), SnO_2_ (110), and RuO_2_ (110), respectively, confirming the composition of the synthesized catalyst as Nb_2_O_5_, SnO_2_, and RuO_2_. Moreover, the clear lattice fringe observed in the SnO_2_/Nb_2_O_5_@RuO_2_ suggests the formation of a well-crystallized degree. The HAADF-STEM and EDX elemental analyses shown in Figure 1d–h indicate highly overlapping signals for Sn, Nb, Ru, and O elements, affirming the intimate contact between Nb_2_O_5_, SnO_2_, and RuO_2_ components within the SnO_2_/Nb_2_O_5_@RuO_2_ catalyst. Using TEM coupled with EDX analysis (Figure 1i) and characterization by inductively coupled plasma mass spectrometry (ICP-MS), it was determined that Nb_2_O_5_ and RuO_2_ comprised approximately 3 wt% and 2 wt%, respectively, in the SnO_2_/Nb_2_O_5_@RuO_2_ system (Table S1).

**Figure 2:**
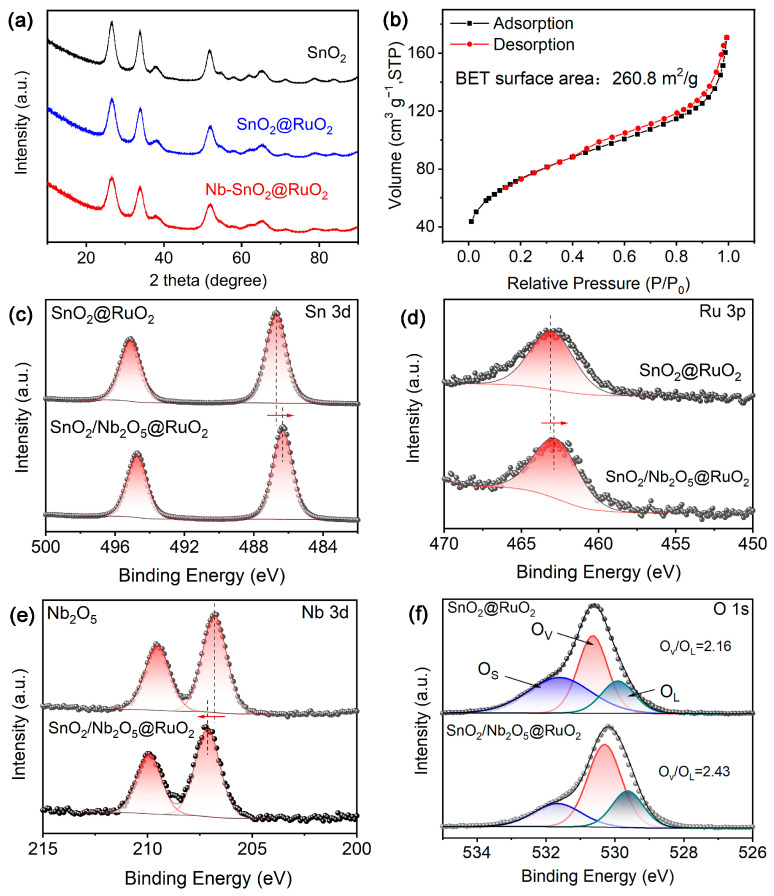
(**a**) XRD patterns of SnO_2_, SnO_2_@RuO_2_, and SnO_2_/Nb_2_O_5_@RuO_2_; (**b**) nitrogen adsorption–desorption analysis of SnO_2_/Nb_2_O_5_@RuO_2_; (**c**–**f**) XPS spectra of Sn 3d, Ru 3p, Nb 3d, and O 1 s regions of SnO_2_@RuO_2_ and SnO_2_/Nb_2_O_5_@RuO_2_.

By analyzing the X-ray diffraction patterns (XRD) of as-prepared samples in Figure 2a, composition information can be obtained. SnO_2_ and SnO_2_@RuO_2_ were synthesized using the same method as reference materials. Apparently, all samples exhibit nearly identical characteristic diffraction peaks, which perfectly match the PDF for SnO_2_ (PDF 00-001-0657) [23]. Unfortunately, diffraction peaks for Nb_2_O_5_ and RuO_2_ materials were not detected, likely due to their low concentrations falling below the detection limit of XRD (ICP: ~3 wt%, ~2 wt%). For those samples depicted in Figure 2b, the BET surface area for SnO_2_/Nb_2_O_5_@RuO_2_ was up to 260 m^2^ g^−1^, with an average adsorption pore size of approximately 0.4 nm (Figure S1), which was slightly larger than that of the SnO_2_ (226.7 m^2^ g^−1^) and SnO_2_@RuO_2_ (241.6 m^2^ g^−1^) (Figure S2). This increase in BET surface area for SnO_2_/Nb_2_O_5_@RuO_2_ enabled a broader contact interface between RuO_2_ and the electrolyte, thereby improving both mass and charge transfer processes within the prepared sample. Typically, X-ray photoelectron spectroscopy (XPS) was utilized to analyze the surface elemental composition of the as-prepared samples. Figure S3 shows the XPS survey spectra of SnO_2_/Nb_2_O_5_@RuO_2_ and SnO_2_@RuO_2_, respectively. In cases of SnO_2_/Nb_2_O_5_@RuO_2_ and SnO_2_@RuO_2_ catalysts, the Nb 3d, Sn 3d, and Ru 3p spectra show peaks corresponding to their respective oxidation states: Nb in the +5 state, Sn in the +2 state, and Ru in the +4 state (Figure 2c–e) [24,25,26], suggesting the successful introduction of Nb_2_O_5_ into SnO_2_@RuO_2_. Notably, introducing Nb_2_O_5_ in the SnO_2_/Nb_2_O_5_@RuO_2_ catalyst induces significant electronic interactions at the interfaces. Specifically, the Sn 3d and Ru 3p spectra exhibit a negative shift of approximately 0.4 eV, indicating a transfer of electrons within the catalyst structure [27]. Conversely, the Nb 3d spectrum shifts to higher energy levels compared to pristine Nb_2_O_5_, suggesting a change in the electronic environment of Nb upon integration into the composite. In simpler terms, electrons from the Nb surface migrate toward the surfaces of Ru and Sn in the SnO_2_/Nb_2_O_5_@RuO_2_ catalyst, resulting in Ru adopting an electron-rich state. Importantly, such an electron-rich environment around RuO_2_ reduces the propensity for forming high-valent ruthenium species [16], which are more susceptible to dissolution issues and contribute significantly to the catalyst’s enhanced stability during long-term tests. In addition, the peaks of lattice oxygen (O_L_) at 529.7 eV, defect-related oxygen (O_d_) at 530.3 eV, and surface-adsorbed oxygen (O_S_) at 531.7 eV can belong to O 1 s (Figure 2f) [28,29]. It has been reported that the existence of oxygen vacancy can produce unsaturated coordination at the metal site, avoiding the involvement of lattice oxygen, and thereby inducing lattice oxygen mediated mechanism-oxygen vacancy site mechanism (LOM-OVSM) [30], improving the stability and activity. Analysis of the oxygen content in Figure 2f and Table S2 shows that the proportion of oxygen vacancy sites and lattice oxygen (O_d_/O_L_) increased from 2.16 to 2.42 after the introduction of Nb_2_O_5_. The literature-reported LOM-OVSM mechanism will likely occur at such high-oxygen-vacancy concentrations.

### 3.2. Electrochemical Analysis

**Figure 3:**
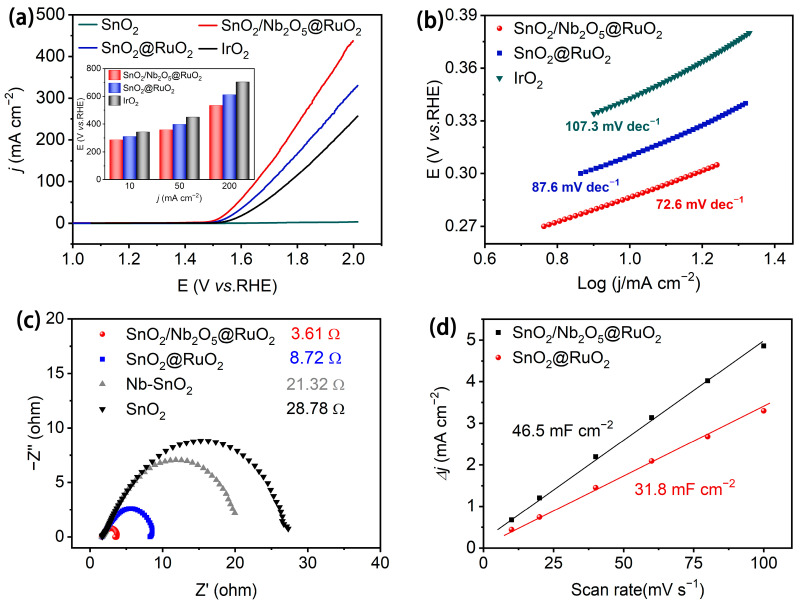
(**a**) Polarization curves of SnO_2_, SnO_2_@RuO_2_, SnO_2_/Nb_2_O_5_@RuO_2_, and commercial IrO_2_. The insert is the overpotential at η_10_, η_50_, and η_200_, respectively. (**b**) The corresponding Tafel plots of SnO_2_@RuO_2_ and SnO_2_/Nb_2_O_5_@RuO_2_ and commercial IrO_2_. (**c**) The Nyquist plots for SnO_2_, Nb_2_O_5_/SnO_2_, SnO_2_@RuO_2_, and SnO_2_/Nb_2_O_5_@RuO_2_; (**d**) The capacitive currents (∆j = j_a_ − j_c_) as a function of scan rate for SnO_2_@RuO_2_ and SnO_2_/Nb_2_O_5_@RuO_2_.

The electrochemical performance of the catalyst for the oxygen evolution reaction (OER) was evaluated using a standard three-electrode cell setup. Polarization current–voltage curves were measured for commercial IrO_2_, SnO_2_, SnO_2_@RuO_2_, and SnO_2_/Nb_2_O_5_@RuO_2_ in an acidic testing solution at a scan rate of 5 mV s^−1^, with 95% iR correction applied to the recorded data (Figure 3a). The inset illustrates the overpotential values of these samples at current densities of 10 mA/cm^2^ (η_10_), 50 mA/cm^2^ (η_50_), and 100 mA/cm^2^ (η_100_). SnO_2_/Nb_2_O_5_@RuO_2_ showed overpotential values of 287 mV, 359 mV, and 534 mV for η_10_, η_50_, and η_100_, respectively, which were much lower than those of SnO_2_@RuO_2_ and commercial IrO_2_. Additionally, the catalytic kinetics of the catalysts in the OER were analyzed through Tafel slope measurements. As shown in Figure 3b, the Tafel slope of SnO_2_/Nb_2_O_5_@RuO_2_ was 72 mV dec^−1^, significantly lower than that of commercial IrO_2_ (107.3 mV dec^−1^) and SnO_2_@RuO_2_ (87.6 mV dec^−1^), indicating enhanced OER kinetics facilitated by Nb_2_O_5_. Furthermore, SnO_2_/Nb_2_O_5_@RuO_2_ exhibited a reduced impedance compared to SnO_2_ (28.78 Ω), SnO_2_@RuO_2_ (8.72 Ω), and Nb_2_O_5_/SnO_2_ (21.32 Ω) (Figure 3c), indicating that the introduction of Nb_2_O_5_ effectively enhances the mass and charge transfer processes in the OER reaction. The electrochemical surface area (ECSA) serves as a direct measure of the active catalytic surface area during electrocatalytic reactions. By performing cyclic voltammetry at various scan rates (Figure S4) and analyzing the relationship between capacitance current and scan rate, the double-layer capacitance (C_dl_) values were determined to be 31.8 mF cm^−2^ for SnO_2_@RuO_2_ and 46.5 mF cm^−2^ for SnO_2_/Nb_2_O_5_@RuO_2_, respectively (Figure 3d). Calculations based on ECSA = C_dl_/C_s_ [31] revealed an ECSA of 1162.5 cm^2^ for SnO_2_/Nb_2_O_5_@RuO_2_ (Table S3), indicating a significantly larger electrochemically active surface area with more active sites compared to the other samples. In conclusion, SnO_2_/Nb_2_O_5_@RuO_2_ demonstrates superior electrochemical performance for the OER, characterized by lower overpotentials, a lower Tafel slope, indicating faster kinetic processes, enhanced mass, and charge transfer, and a significantly larger ECSA, all of which contribute to its exceptional catalytic activity in acidic environments.

**Figure 4:**
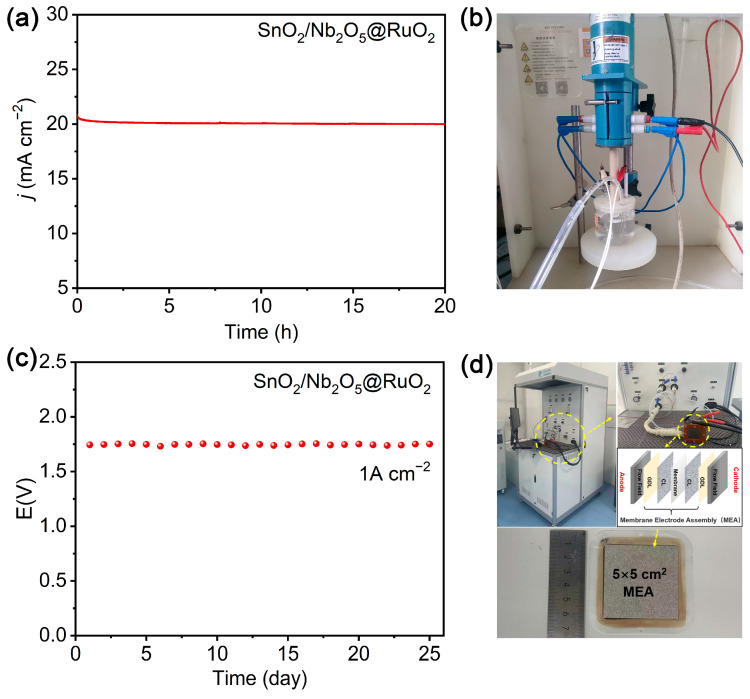
**(a**) The chronopotentiometry stability measurement of SnO_2_/Nb_2_O_5_@RuO_2_ in the three-electrode system at a current density of 10 mA cm^−2^. (**b**) A schematic diagram of the three-electrode water splitting device. (**c**) The chronopotentiometry stability measurement of SnO_2_/Nb_2_O_5_@RuO_2_ in the PEMWE system. (**d**) A schematic diagram of the PEMWE device.

Typically, the catalytic stability of PEM catalysts is a critical parameter. Initial testing was conducted using i–t (current–time) mode, where SnO_2_/Nb_2_O_5_@RuO_2_ showed minimal potential fluctuation over 20 h under a constant current density of 20 mA cm^−2^ in a three-electrode system (Figure 4a,b). Furthermore, in a practical application setup resembling a PEMWE system, SnO_2_/Nb_2_O_5_@RuO_2_ was employed as the anode, while commercial Pt/C catalysts served as the cathode. As displayed in Figure 4c, the system required a cell potential of approximately 1.75 V in 0.5 M H_2_SO_4_ electrolyte to achieve a current density of 1 A cm^−2^ in a PEMWE test bench (Figure 4d). Notably, the SnO_2_/Nb_2_O_5_@RuO_2_ catalyst demonstrated stable operation for 25 days under constant current density conditions, reaching the basic industrial application level. In summary, the SnO_2_/Nb_2_O_5_@RuO_2_ catalysts show excellent electrochemical stability and performance in both laboratory settings and practical PEMWE applications, highlighting their potential for use in efficient and long-lasting hydrogen production systems. To further demonstrate the stability of the catalyst, TEM, SEM, and XRD characterization were performed on SnO_2_/Nb_2_O_5_@RuO_2_. As shown in Figures S5–S7, the morphology and component of the SnO_2_/Nb_2_O_5_@RuO_2_ catalyst did not change significantly before and after i–t stability testing, indicating that SnO_2_/Nb_2_O_5_@RuO_2_ had excellent catalytic durability. In summary, our prepared catalyst exhibited excellent activity and stability, and its combined performance exceeded that of previously reported partial IR-based and Ru-based materials (Table S4).

### 3.3. Mechanism Analysis

**Figure 5:**
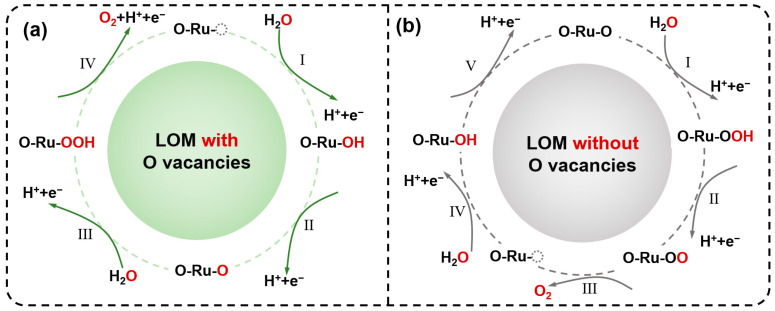
(**a**) The LOM-OVSM mechanism of acid OER process on SnO_2_/Nb_2_O_5_@RuO_2_. (**b**) LOM mechanism of acid OER process on conventional RuO_2_.

Building upon the exceptional catalytic performance and detailed characterization of SnO_2_/Nb_2_O_5_@RuO_2_, we delve deeper into elucidating the OER mechanism. As shown in Figure 5a, the adsorption configuration of oxygen-containing intermediates on the surface of RuO_2_ in SnO_2_/Nb_2_O_5_@RuO_2_ follows the LOM-OVSM mechanism (*→*OH→*O→*OOH→O_2_) [30]. Detailly, the presence of O vacancies allows *OH generated from H_2_O to fill these vacancies, and through four consecutive proton–electron transfer processes, H_2_O molecules are ultimately converted into O_2_. The entire process avoids the participation of lattice oxygen and, thus, significantly improves the stability. In contrast, the RuO_2_ without oxygen vacancies undergoes the typical LOM mechanism. Importantly, the generated *O can be coupled with the lattice oxygen in RuO_2_ to form and release O_2_, creating oxygen defects that cause RuO_2_ to dissolve, significantly affecting its stability (Figure 5b). Simultaneously, in SnO_2_/Nb_2_O_5_@RuO_2_, the high electron density on the Nb_2_O_5_ surface facilitates its migration towards the RuO_2_ and SnO_2_ surface, thereby preventing Ru from forming high-valent ruthenium species, further inhibiting its dissolution. Consequently, these measures notably enhance the catalyst’s activity and durability.

## 4. Conclusions

In summary, we employed a straightforward approach to fabricate a SnO_2_, Nb_2_O_5_, and RuO_2_ composite catalyst (SnO_2_/Nb_2_O_5_@RuO_2_) featuring multiple interfaces and a high concentration of oxygen vacancies. Incorporating Nb_2_O_5_ in the SnO_2_/Nb_2_O_5_@RuO_2_ catalyst modifies the electronic structure at the interfaces and induces rich oxygen vacancy, inhibiting RuO_2_ dissolution and, thus, enhancing durability. In addition, due to its large surface area and abundant active sites, the SnO_2_/Nb_2_O_5_@RuO_2_ catalyst exhibits outstanding acidic oxygen evolution reaction (OER) activity. Consequently, the Nb-SnO_2_@RuO_2_ catalyst achieves current densities of 10, 50, and 200 mA cm^−2^ at ultralow overpotentials of 287, 359, and 534 mV, respectively, which are much lower than those of commercial IrO_2_. Importantly, the membrane electrode composed of SnO_2_/Nb_2_O_5_@RuO_2_ and commercial Pt/C was run stably in the PEM cell for 25 days at industrial current density (1 A cm^−2^). This work provides an easy method for developing a highly efficient and durable Ru-based electrocatalyst, showing its potential for PEM water electrolysis.

## Data Availability

The original contributions presented in the study are included in the article/Supplementary Materials, further inquiries can be directed to the corresponding authors.

## Supplementary Materials

The following supporting information can be downloaded at: https://www.mdpi.com/article/10.3390/ma17204991/s1. Figure S1. The pore size distribution of SnO_2_/Nb_2_O_5_@RuO_2_. Figure S2. N_2_ adsorption/desorption isotherms of SnO_2_ and SnO_2_@RuO_2_. Figure S3. XPS survey spectra of SnO_2_@RuO_2_ and SnO_2_/Nb_2_O_5_@RuO_2_. Figure S4. CV curves were performed at various scan rates in the region of −0.2 V to −0.1 V (vs. SCE) for (a) SnO_2_/Nb_2_O_5_@RuO_2_ and (b) SnO_2_@RuO_2_. Figure S5. SEM images of SnO_2_/Nb_2_O_5_@RuO_2_ before (a–c) and after (d–f) i–t test. Figure S6. (a) TEM image and (b) HAADF-STEM image of SnO_2_/Nb_2_O_5_@RuO_2_ after i–t test; (c–f) the elemental mappings of Sn, Nb, Ru, and O for SnO_2_/Nb_2_O_5_@RuO_2_ after i–t test. Figure S7. XRD patterns of SnO_2_/Nb_2_O_5_@RuO_2_ before and after i–t test. Table S1. The ICP-MS data of SnO_2_/Nb_2_O_5_@RuO_2_. Table S2. The O 1s regions for SnO_2_@RuO_2_ and SnO_2_/Nb_2_O_5_@RuO_2_. Table S3. The ECSA values of SnO_2_@RuO_2_ and SnO_2_/Nb_2_O_5_@RuO_2_, respectively. Table S4. Comparison of SnO_2_/Nb_2_O_5_@RuO_2_ with other Ir-, Ru-based catalysts in terms of OER performance in 0.5 M H_2_SO_4_ electrolyte.

## References

[1] Obi M., Bass R. (2016). Trends and challenges of grid-connected photovoltaic systems—A review. Renew. Sustain. Energy Rev..

[2] Chen Z., Guo L., Pan L., Yan T., He Z., Li Y., Shi C., Huang Z.F., Zhang X., Zou J.J. (2022). Advances in Oxygen Evolution Electrocatalysts for Proton Exchange Membrane Water Electrolyzers. Adv. Energy Mater..

[3] Kumar S.S., Himabindu V. (2019). Himabindu, Hydrogen production by PEM water electrolysis—A review. Mater. Sci. Energy Technol..

[4] Song J., Wei C., Huang Z.F., Liu C., Zeng L., Wang X., Xu Z.J. (2020). A review on fundamentals for designing oxygen evolution electrocatalysts. Chem. Soc. Rev..

[5] Wen Y., Chen P., Wang L., Li S., Wang Z., Abed J., Mao X., Min Y., Dinh C.T., De Luna P. (2021). Sargent, Stabilizing Highly Active Ru Sites by Suppressing Lattice Oxygen Participation in Acidic Water Oxidation. J. Am. Chem. Soc..

[6] She L., Zhao G., Ma T., Chen J., Sun W., Pan H. (2022). On the Durability of Iridium-Based Electrocatalysts toward the Oxygen Evolution Reaction under Acid Environment. Adv. Funct. Mater..

[7] Shirvanian P., van Berkel F. (2020). Novel components in Proton Exchange Membrane (PEM) Water Electrolyzers (PEMWE): Status, challenges and future needs. A mini review. Electrochem. Commun..

[8] Liu G., Hou F., Wang X., Fang B. (2023). Ir-IrO_2_ with heterogeneous interfaces and oxygen vacancies-rich surfaces for highly efficient oxygen evolution reaction. Appl. Surf. Sci..

[9] Higashi S., Beniya A. (2023). Ultralight conductive IrO_2_ nanostructured textile enables highly efficient hydrogen and oxygen evolution reaction: Importance of catalyst layer sheet resistance. Appl. Catal. B Environ..

[10] Chen F.-Y., Wu Z.-Y., Adler Z., Wang H. (2021). Stability challenges of electrocatalytic oxygen evolution reaction: From mechanistic understanding to reactor design. Joule.

[11] Ping X., Liu Y., Zheng L., Song Y., Guo L., Chen S., Wei Z. (2024). Locking the lattice oxygen in RuO_2_ to stabilize highly active Ru sites in acidic water oxidation. Nat. Commun..

[12] Hao S., Liu M., Pan J., Liu X., Tan X., Xu N., He Y., Lei L., Zhang X. (2020). Dopants fixation of Ruthenium for boosting acidic oxygen evolution stability and activity. Nat. Commun..

[13] Wu Y., Yao R., Zhang K., Zhao Q., Li J., Liu G. (2024). RuO_2_/CeO_2_ heterostructure anchored on carbon spheres as a bifunctional electrocatalyst for efficient water splitting in acidic media. Chem. Eng. J..

[14] Wu Z., Wang Y., Liu D., Zhou B., Yang P., Liu R., Xiao W., Ma T., Wang J., Wang L. (2023). Hexagonal Defect-Rich MnO/RuO_2_ with Abundant Heterointerface to Modulate Electronic Structure for Acidic Oxygen Evolution Reaction. Adv. Funct. Mater..

[15] Du K., Zhang L., Shan J., Guo J., Mao J., Yang C.-C., Wang C.-H., Hu Z., Ling T. (2022). Interface engineering breaks both stability and activity limits of RuO_2_ for sustainable water oxidation. Nat. Commun..

[16] Li J., Yao M., Yuan Z., Ma J., Geng S., Liu F. (2024). Phase engineering boosting heterogeneous interface effect in RuO_2_/MnO_2_ catalysts for acidic oxygen evolution reaction. Chem. Eng. J..

[17] Prestat M. (2023). Corrosion of structural components of proton exchange membrane water electrolyzer anodes: A review. J. Power Sources.

[18] Jin M., Zhang X., Niu S., Wang Q., Huang R., Ling R., Huang J., Shi R., Amini A., Cheng C. (2022). Strategies for Designing High-Performance Hydrogen Evolution Reaction Electrocatalysts at Large Current Densities above 1000 mA cm^–2^. ACS Nano.

[19] Luo Y., Zhang Z., Chhowalla M., Liu B. (2022). Recent Advances in Design of Electrocatalysts for High-Current-Density Water Splitting. Adv. Mater..

[20] Su K., Liu H., Gao Z., Fornasiero P., Wang F. (2021). Nb_2_O_5_-Based Photocatalysts. Adv. Sci..

[21] Deng B., Lei T., Zhu W., Xiao L., Liu J. (2018). In-Plane Assembled Orthorhombic Nb_2_O_5_ Nanorod Films with High-Rate Li^+^ Intercalation for High-Performance Flexible Li-Ion Capacitors. Adv. Funct. Mater..

[22] Burstein G.T. (2005). A hundred years of Tafel’s Equation: 1905–2005. Corros. Sci..

[23] Haq S., Sarfraz A., Menaa F., Shahzad N., Din S.U., Almukhlifi H.A., Alshareef S.A., Al Essa E.M., Shahzad M.I. (2022). Green Synthesis of NiO-SnO_2_ Nanocomposite and Effect of Calcination Temperature on Its Physicochemical Properties: Impact on the Photocatalytic Degradation of Methyl Orange. Molecules.

[24] Yang H., He F., Shen J., Chen Z., Yao Y., He L., Yu Y. (2023). 2D Nb_2_O_5_@2D metallic RuO_2_ heterostructures as highly reversible anode materials for lithium-ion batteries. Energy Lab..

[25] Liu L., Wang Y., Guan K., Liu Y., Li Y., Sun F., Wang X., Zhang C., Feng S., Zhang T. (2023). Influence of oxygen vacancies on the performance of SnO_2_ gas sensing by near-ambient pressure XPS studies. Sens. Actuators B Chem..

[26] Cong N., Han Y., Tan L., Zhai C., Chen H., Han J., Fang H., Zhou X., Zhu Y., Ren Z. (2021). Nanoporous RuO_2_ characterized by RuO(OH)_2_ surface phase as an efficient bifunctional catalyst for overall water splitting in alkaline solution. J. Electroanal. Chem..

[27] Liu L., Wang Z., Zhang J., Ruzimuradov O., Dai K., Low J. (2023). Tunable Interfacial Charge Transfer in a 2D–2D Composite for Efficient Visible-Light-Driven CO2 Conversion. Adv. Mater..

[28] Kuang M., Wang Y., Fang W., Tan H., Chen M., Yao J., Liu C., Xu J., Zhou K., Yan Q. (2020). Efficient Nitrate Synthesis via Ambient Nitrogen Oxidation with Ru-Doped TiO_2_/RuO_2_ Electrocatalysts. Adv. Mater..

[29] Cong Y., Tang Q., Wang X., Liu M., Liu J., Geng Z., Cao R., Zhang X., Zhang W., Huang K. (2019). Silver-Intermediated Perovskite La_0.9_FeO_3−δ_ toward High-Performance Cathode Catalysts for Nonaqueous Lithium–Oxygen Batteries. ACS Catal..

[30] Wang Y., Yang R., Ding Y., Zhang B., Li H., Bai B., Li M., Cui Y., Xiao J., Wu Z.-S. (2023). Unraveling oxygen vacancy site mechanism of Rh-doped RuO_2_ catalyst for long-lasting acidic water oxidation. Nat. Commun..

[31] Huang Y., Gong Q., Song X., Feng K., Nie K., Zhao F., Wang Y., Zeng M., Zhong J., Li Y. (2016). Mo2C Nanoparticles Dispersed on Hierarchical Carbon Microflowers for Efficient Electrocatalytic Hydrogen Evolution. ACS Nano.

