# Supplementary material for: Interface-Strengthened Ru-Based Electrocatalyst for High-Efficiency Proton Exchange Membrane Water Electrolysis at Industrial-Level Current Density
Source: Materials (Basel). 2024 Oct 12;17(20):4991. doi: 10.3390/ma17204991 (PMC11509503; doi:10.3390/ma17204991)
Supplement: Supplementary file 1 [file materials-17-04991-s001.zip › materials-3212060-supplementary.pdf]

[Title]

Interface-Strengthened Ru-Based Electrocatalyst for High-Efficiency Proton Exchange Membrane Water Electrolysis at Industrial-Level Current Density

Wenjun Lei[1], Xinxin Zhao[1], Chao Liang[2], Huai Wang[2], Xuehong Li[1], Mingkun Jiang[1], Xiaofeng Li[1], Fengqin He[1], Yonghui Sun[1], Gang Lu[*,1], Hairui Cai[*,2]

1. Qinghai Upstream of the Yellow River Hydropower Development Co., Ltd. Photovoltaic Industry Technology Branch Company, State Power Investment Corporation, Photovoltaic (Energy Storage) Industry Innovation Center, Photovoltaic Technology Research and Development Department, No. 399 South Yanta Road, Xi'an, 710000, China
2. MOE Key Laboratory for Non-Equilibrium Synthesis and Modulation of Condensed Matter, Key Laboratory of Shaanxi for Advanced Materials and Mesoscopic Physics, State Key Laboratory for Mechanical Behavior of Materials, School of Physics, Xi’an Jiaotong University, No. 28 West Xianning Road, Xi'an 710049, China

Correspondence: eelugang@163.com (G.L.); caihairui@xjtu.edu.cn (H.C.)

Characterization.

The crystalline structure of as-prepared samples was analyzed by X-ray powder diffraction (XRD, Bruker D8 Advance X-ray diffractometer using Cu Kα radiation (λ = 0.15418 nm), Germany). The morphology was studied by scanning electron microscopy (SEM, JEOL JSM-7000F) and transmission electron microscopy (TEM, JEOL JEM-F200). The chemical states of samples were characterized by Thermo Fisher X-ray photoelectron spectroscopy (XPS, ESCALAB Xi+) with Mg Kα as the excitation source ($h\nu$ = 1253.69 eV), and the standard C 1s peak is 284.8 eV.

Table S1. The ICP-MS data of $SnO_2/Nb_2O_5@RuO_2$.

**Catalyst**	**Nb**<br>**（ug/L）**	**Ru**<br>**（ug/L）**	**Sn**<br>**（ug/L）**
**$SnO_2/Nb_2O_5@RuO_2$**	120.91	133.90	4376

Table S2. The O 1s regions for $SnO_2@RuO_2$ and $SnO_2/Nb_2O_5@RuO_2$.

Catalyst	O species	Peak BE	Area CPS	O_d concentration	O_d/O_L
$SnO_2@RuO_2$	O_d	530.64	34330.1	68%	2.16
	O_S	531.61	33626.41		
	O_L	529.91	15905.62		
$SnO_2/Nb_2O_5@RuO_2$	O_d	530.3	41534.11	71%	2.43
	O_S	531.67	16527.28		
	O_L	529.63	17106.94		

Table S3. The ECSA values of SnO_2@RuO_2 and SnO_2/Nb_2O_5@RuO_2, respectively.

Catalyst	C_{dl} （mF cm^{-2}）	C_s （mF cm^{-2}）	ECSA (cm^2)
**SnO_2@RuO_2**	31.8	0.04	795
**SnO_2/Nb_2O_5@RuO_2**	46.5	0.04	1162.5

Table S4. Comparison of $SnO_2/Nb_2O_5@RuO_2$ with other Ir-, Ru-based catalysts in terms of OER performance in 0.5 M H_2SO_4 electrolyte.

Catalyst	η_{10} (mV)	Stability	Ref.
**$SnO_2/Nb_2O_5@RuO_2$**	**287**	**25 day@1 Acm^{-2}**	**This work**
**$Ta\text{-}RuO_2$**	201	280 h@10 mA cm^{-2}	[1]
**$RuNiO_x$**	217	100 h@10 mA cm^{-2}	[2]
**$Ru/RuO_2\text{-}Co_3O_4$**	226	20 h@10 mA cm^{-2}	[3]
**RuO_2-TiO2**	238	30 h@10 mA cm^{-2}	[4]
**$WO_3@RuO_2$**	246	50 h@10 mA cm^{-2}	[5]
**$Ru_{0.6}W_{17.4}O_{49-\delta}$**	252	45 h@10 mA cm^{-2}	[6]
**$NaRuO_2$**	260	6 h@10 mA cm^{-2}	[7]
**$Ir@WO_{3-x}$**	276	110 h@1.6V	[8]
**$Ru\text{-}SA/Ti_3C_2T_x$**	290	32 h@10 mA cm^{-2}	[9]
**$Y_{1.85}Zn_{0.15}Ru_2O_{7-x}$**	291	8 h@1 mA cm^{-2}	[10]
**Ir/SiO_2**	305	-	[11]
**$IrRuO_x/TiO_2$**	320	100 th@CV cycle	[12]
**$Ir@WO_xNR_s$**	330	1030 h@0.5 A cm^{-2}	[13]
**$Mn/TiO_2\text{-}RuO_2$**	386	6 h@1 mA cm^{-2}	[14]

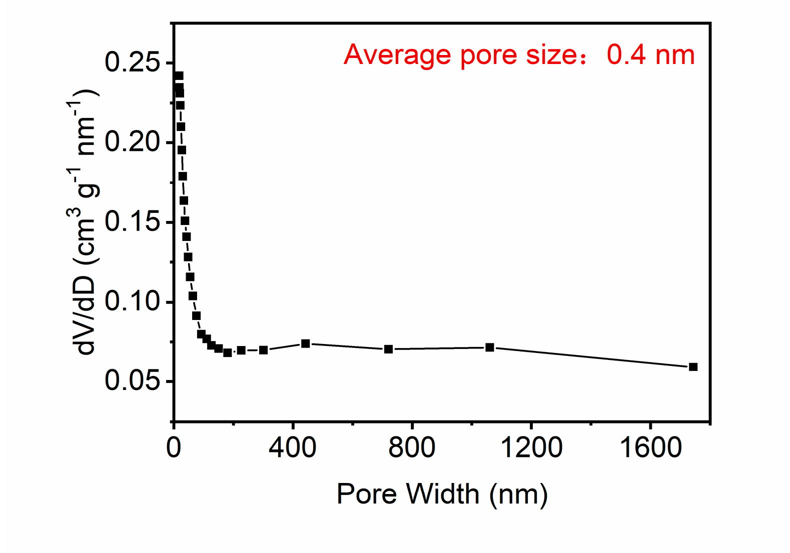

Figure S1 The pore size distribution of $SnO_2/Nb_2O_5@RuO_2$

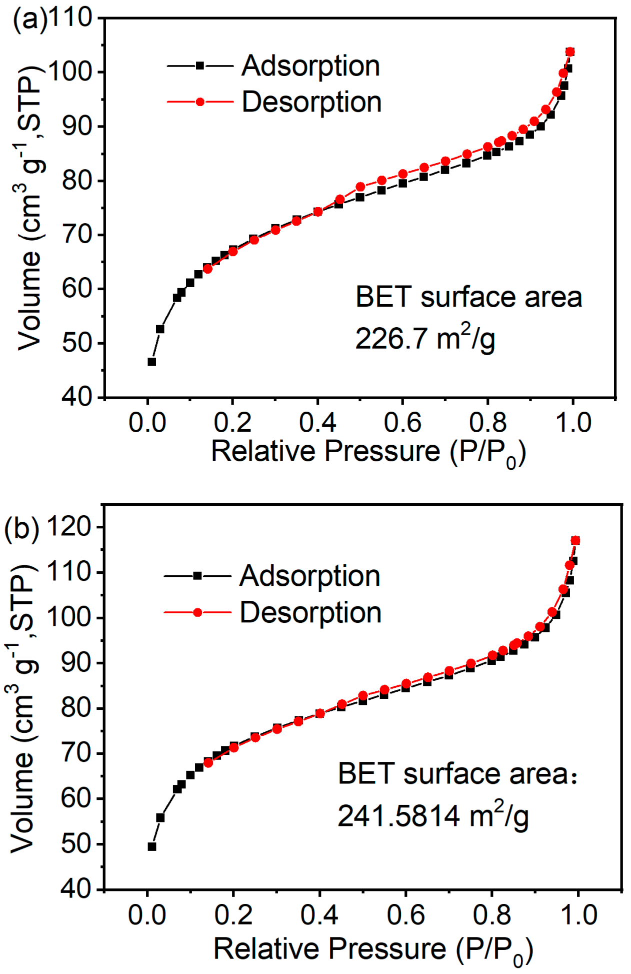

Figure S2 N_2 adsorption/desorption isotherms of SnO_2 and SnO_2@RuO_2

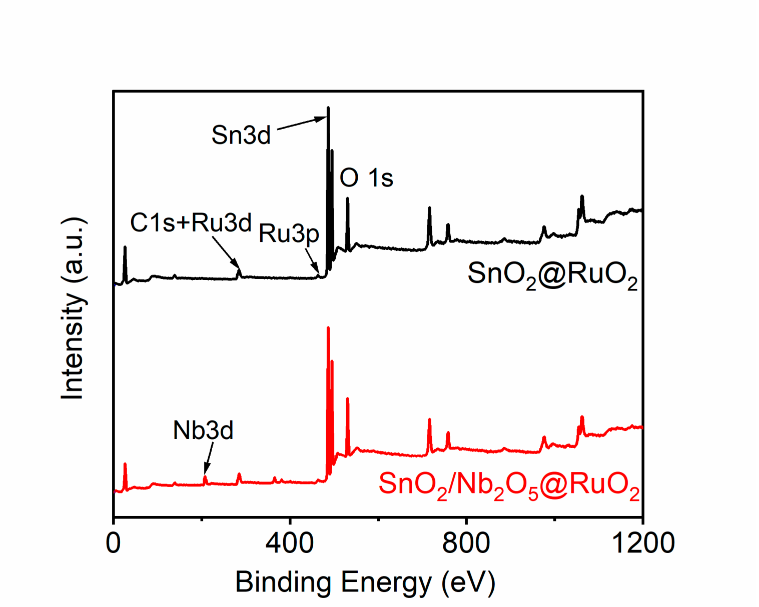

Figure S3. XPS survey spectra of SnO_2@RuO_2 and SnO_2/Nb_2O_5@RuO_2.

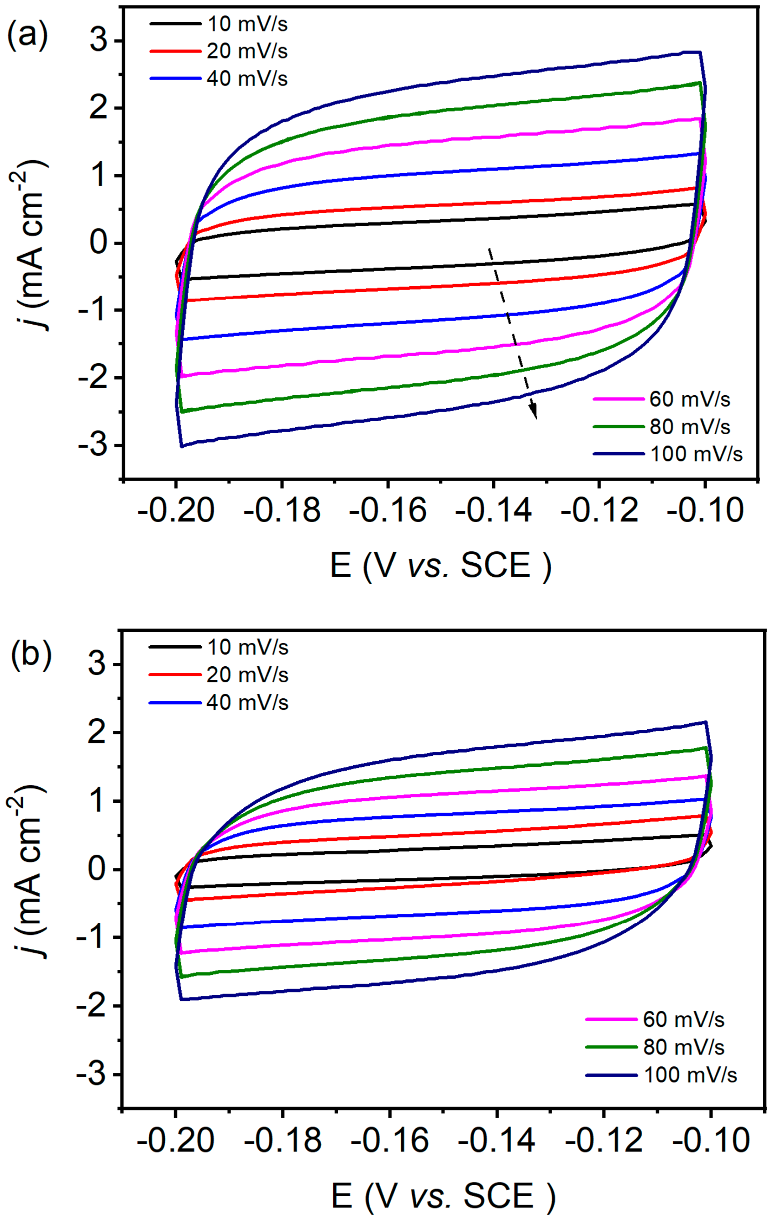

Figure S4. CV curves were performed at various scan rates in the region of -0.2 V to -0.1 V (*vs.* SCE) for (a) $SnO_2/Nb_2O_5@RuO_2$ and (b) $SnO_2@RuO_2$.

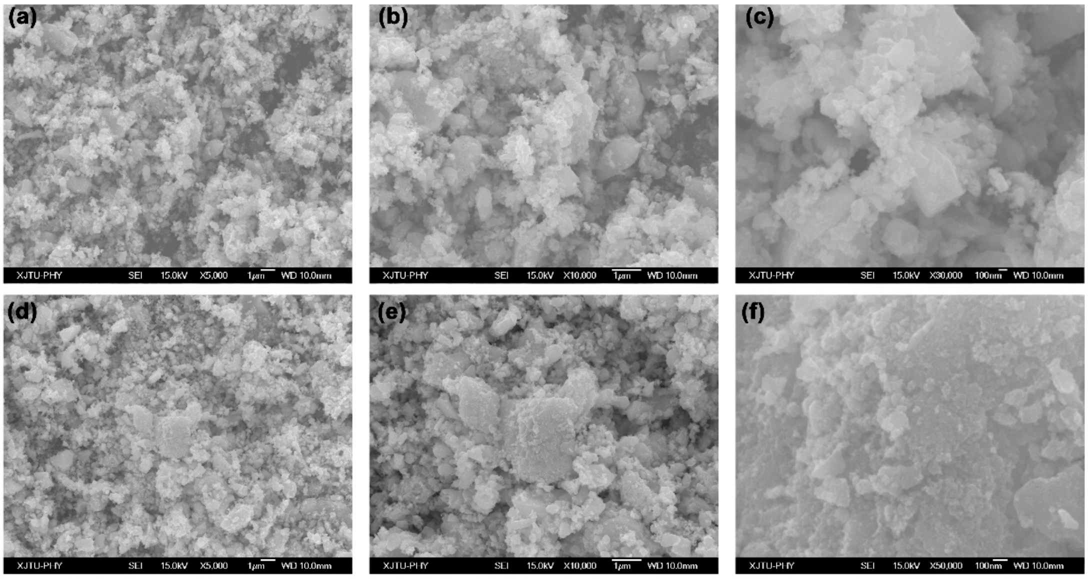

Figure S5. SEM images of $SnO_2/Nb_2O_5@RuO_2$ before (a-c) and after (d-f) i–t test.

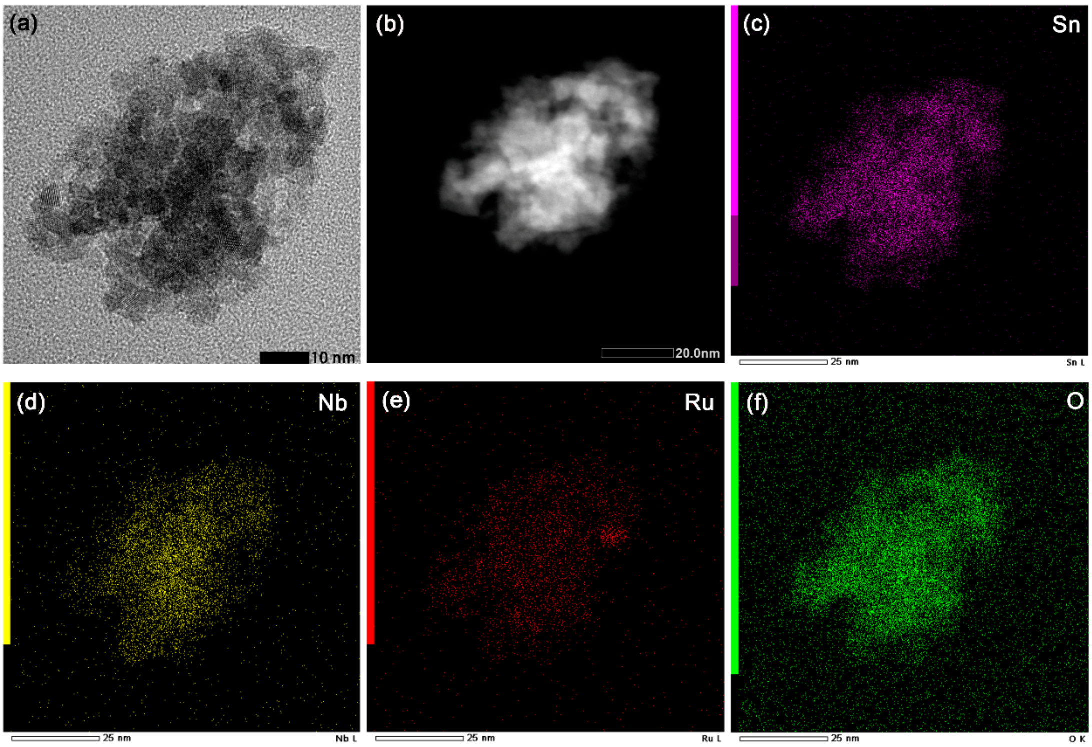

Figure S6. (a) TEM image and (b) HAADF-STEM image of $SnO_2/Nb_2O_5@RuO_2$ after i–t test; (c–f) the elemental mappings of Sn, Nb, Ru, and O for $SnO_2/Nb_2O_5@RuO_2$ after i–t test.

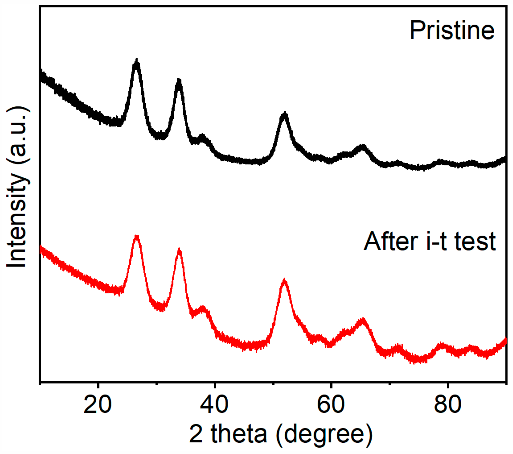

Figure S7. XRD patterns of $SnO_2/Nb_2O_5@RuO_2$ before and after i–t test.

[1] X. Wang, Z. Li, H. Jang, C. Chen, S. Liu, L. Wang, M.G. Kim, J. Cho, Q. Qin, X. Liu, RuO2 with Short-Range Ordered Tantalum Single Atoms for Enhanced Acidic Oxygen Evolution Reaction, Advanced Energy Materials, n/a (2024) 2403388.
[2] Y.-J. Ko, M.H. Han, C. Lim, S.-H. Yu, C.H. Choi, B.K. Min, J.-Y. Choi, W.H. Lee, H.-S. Oh, Unveiling the role of Ni in Ru-Ni oxide for oxygen evolution: Lattice oxygen participation enhanced by structural distortion, Journal of Energy Chemistry, 77 (2023) 54-61.
[3] T. Wang, Z. Li, H. Jang, M.G. Kim, Q. Qin, X. Liu, Interface Engineering of Oxygen Vacancy-Enriched Ru/RuO2–Co3O4 Heterojunction for Efficient Oxygen Evolution Reaction in Acidic Media, ACS Sustainable Chemistry & Engineering, 11 (2023) 5155-5163.
[4] J. Zhang, Y. Song, W. Liu, Q. Zheng, Y. Liu, T. Wu, T. Li, Enhancing the acidic oxygen evolution reaction performance of RuO2-TiO2 by a reduction-oxidation process, Nanotechnology, 35 (2024) 345703.
[5] X. Zhang, F. Wu, Q. Zhang, Z. Lu, Y. Zheng, Y.a. Zhu, Y. Lin, Self-Supported WO3@RuO2 Nanowires for Electrocatalytic Acidic Water Oxidation, Inorganic Chemistry, 63 (2024) 8418-8425.
[6] X. Wang, H. Jang, S. Liu, Z. Li, X. Zhao, Y. Chen, M.G. Kim, Q. Qin, X. Liu, Enhancing the Catalytic Kinetics and Stability of Ru Sites for Acidic Water Oxidation by Forming Brønsted Acid Sites in Tungsten Oxide Matrix, Advanced Energy Materials, 13 (2023) 2301673.
[7] S. Laha, Y. Lee, F. Podjaski, D. Weber, V. Duppel, L.M. Schoop, F. Pielnhofer, C. Scheurer, K. Müller, U. Starke, K. Reuter, B.V. Lotsch, Ruthenium Oxide Nanosheets for Enhanced Oxygen Evolution Catalysis in Acidic Medium, Advanced Energy Materials, 9 (2019) 1803795.
[8] X. Ma, C. Yang, F. Zhang, F. Ke, Q. Cheng, L. Zou, H. Yang, Oxygen-vacancy-rich tungsten oxide boosted ultrasmall iridium nanoparticles for acidic oxygen evolution, International Journal of Hydrogen Energy, 48 (2023) 36776-36783.
[9] X. Peng, S. Zhao, Y. Mi, L. Han, X. Liu, D. Qi, J. Sun, Y. Liu, H. Bao, L. Zhuo, H.L. Xin, J. Luo, X. Sun, Trifunctional Single-Atomic Ru Sites Enable Efficient Overall Water Splitting and Oxygen Reduction in Acidic Media, Small, 16 (2020) 2002888.
[10] Q. Feng, Q. Wang, Z. Zhang, Y. Xiong, H. Li, Y. Yao, X.-Z. Yuan, M.C. Williams, M. Gu, H. Chen, H. Li, H. Wang, Highly active and stable ruthenate pyrochlore for enhanced oxygen evolution reaction in acidic medium electrolysis, Applied Catalysis B: Environmental, 244 (2019) 494-501.
[11] Y. Sugita, T. Tamaki, H. Kuroki, T. Yamaguchi, Connected iridium nanoparticle catalysts coated onto silica with high density for oxygen evolution in polymer electrolyte water electrolysis, Nanoscale Advances, 2 (2020) 171-175.
[12] A. Martínez-Séptimo, M.A. Valenzuela, P. Del Angel, R.d.G. González-Huerta, IrRuOx/TiO2 a stable electrocatalyst for the oxygen evolution reaction in acidic media, International Journal of Hydrogen Energy, 46 (2021) 25918-25928.
[13] G. Jiang, H. Yu, Y. Li, D. Yao, J. Chi, S. Sun, Z. Shao, Low-Loading and Highly Stable Membrane Electrode Based on an Ir@WOxNR Ordered Array for PEM Water Electrolysis, ACS Applied Materials & Interfaces, 13 (2021) 15073-15082.
[14] M. Etzi Coller Pascuzzi, A. Goryachev, J.P. Hofmann, E.J.M. Hensen, Mn promotion of rutile TiO2-RuO2 anodes for water oxidation in acidic media, Applied Catalysis B: Environmental, 261 (2020) 118225.
